# Integration of Digital Therapeutics Into Occupational Rehabilitation in Germany: Multilevel Simulation Study

**DOI:** 10.2196/93793

**Published:** 2026-05-26

**Authors:** Christoph Wallner, Sonja Verena Schmidt, Felix Reinkemeier, Dagmar-Maria Dick, Marcus Lehnhardt, Marius Drysch

**Affiliations:** 1 Department for Plastic and Hand Surgery, University Hospital Bergmannsheil Bochum, Bürkle de la Camp-Platz 1, Bochum, 44789, Germany, +49 2343020; 2 BG BAU – German Social Accident Insurance for the Construction Sector, Wuppertal, Germany

**Keywords:** digital therapeutics, digital health applications, simulation modeling, health system capacity, occupational rehabilitation, access to care, cost-consequence analysis

## Abstract

**Background:**

Expenditures for physiotherapy and extended outpatient physiotherapy (EAP) are increasing within Germany’s statutory accident insurance system (Berufsgenossenschaften), placing growing pressure on rehabilitation capacity and timely access to care. Digital health applications (DiGAs) are reimbursable nationwide and represent a novel component of routine rehabilitation pathways. However, their real-world system-level and economic effects in occupational rehabilitation remain insufficiently understood.

**Objective:**

This study aimed to evaluate how the integration of DiGAs into occupational rehabilitation pathways may influence costs, service capacity, and waiting times within routine care delivered by 5 German statutory accident insurance funds that cover 25.9 million insured individuals.

**Methods:**

Aggregated administrative data from 5 Berufsgenossenschaften (fiscal years 2023‐2024) were analyzed using a multilevel simulation framework combining (1) probabilistic cost-consequence modeling with Monte Carlo simulation (10,000 iterations), (2) an adherence-based adoption funnel distinguishing long-term engaged users (15%) and short-term users (85%) based on German claims data, and (3) a calibrated M/M/1 queuing model validated through discrete event simulation to estimate the effects on waiting times and system capacity. Primary outcomes included net financial impact, break-even thresholds, and changes in access-related performance metrics.

**Results:**

Combined physiotherapy and EAP expenditures reached €404 million (€1=US $1.18) in 2024, increasing by 10.1% year-over-year. The primary simulation (N=10,000 iterations) indicated mean annual net savings of €18.4 million (median €17.9 million) with a 90.7% probability of cost savings (95% uncertainty range: net cost of €8 million to net savings of €47.7 million). After incorporating adherence dynamics, the projected mean net savings were €16.2 million (95% CI €5-€29.8 million), corresponding to a 100% probability of positive financial impact within the modeled parameter space. Cost neutrality was maintained for DiGA prices up to €617.8 per prescription, nearly 40% above the base-case assumption of €450, indicating substantial economic robustness. Queuing analyses demonstrated that modest reductions in therapeutic demand decreased mean waiting times from 17.3 to 12.8 days (−26%), equivalent to approximately 120,000 cumulative patient waiting days saved annually across 26,705 EAP patients. The validation of discrete event simulation confirmed the magnitude and direction of analytic estimates.

**Conclusions:**

Under conservative assumptions, integrating digital therapeutics into occupational rehabilitation pathways is likely to generate both economic benefits and substantial system-level capacity gains. The break-even threshold of €617.80 per prescription provides a wide margin for pricing policy. Beyond cost effects, DiGAs may function as scalable capacity tools that alleviate systemic bottlenecks and improve timely access to rehabilitation services in capacity-constrained systems.

## Introduction

### Background and Rationale

The German statutory accident insurance system, managed by the Berufsgenossenschaften, represents a unique institutional framework responsible for the prevention, rehabilitation, and compensation of occupational accidents and diseases for over 85 million insured individuals [1]. Against a backdrop of high and rising expenditures—rehabilitation spending across Germany reached €43.6 billion (€1=US $1.18) in 2022 [2]—traditional therapies such as physiotherapy and extended outpatient physiotherapy (EAP) represent a substantial and growing cost driver [3,4]. Given the scale of the Berufsgenossenschaften system and its ring-fenced financing structure, Germany provides an internationally distinctive setting to assess the system-level impact of novel care models.

A pivotal development in Germany was the establishment of a formal regulated pathway for digital health applications (known by their German acronym, DiGAs) introduced by the 2019 Digital Healthcare Act (Digitale-Versorgung-Gesetz), which enables the prescription and reimbursement of digital therapeutics within routine care [5]. While DiGA uptake has accelerated since the first DiGAs were introduced into the German statutory health insurance system in 2020—cumulative reimbursements reached €234 million by the end of 2024—evaluation has largely emphasized clinical efficacy and patient-relevant structural outcomes [6,7]. A critical evidence gap persists around DiGA’s economic and operational effects in high-volume, high-cost rehabilitation pathways.

### Digital Physiotherapy: International Evidence and the German Context

International studies suggest that digital physiotherapy and telehealth can deliver outcomes comparable to face-to-face care at a lower cost, primarily through reduced resource use and travel [8]; for example, a Swedish analysis reported digital osteoarthritis care at approximately 25% of the cost of in-person care [9]. A large-scale health technology assessment found that virtual musculoskeletal solutions delivered clinically meaningful improvements in pain and function compared with usual care across a range of musculoskeletal disorders [10]. Recent meta-analytic evidence further supports the cost-effectiveness of telerehabilitation relative to traditional in-person rehabilitation [11]. Yet, within Germany, economic evaluations indicate that the DiGA value is sensitive to price and evidentiary strength, with some analyses showing only marginal benefits [12]. To our knowledge, no prior study has quantified the potential cost and capacity effects of DiGA specifically for physiotherapy and EAP within the Berufsgenossenschaften context.

### Study Aim

To address this gap, we developed a multistage quantitative modeling framework integrating probabilistic cost-consequence analysis, claims-based adherence stratification, and calibrated queuing theory to provide a robust, multidimensional assessment of whether, and under what assumptions, DiGA can reduce costs while expanding effective care capacity in Germany’s occupational rehabilitation system. This study, therefore, examines digital therapeutics not only as clinical interventions but also as system-level tools capable of reshaping rehabilitation capacity and access dynamics.

## Methods

### Study Design and Modeling Framework

To evaluate the system-level effects of DiGA integration into occupational rehabilitation pathways, we developed a multilevel simulation framework combining economic, behavioral, and operational modeling approaches. Rather than performing a traditional cost-use evaluation, the study was designed as a system-level cost-consequence and implementation simulation assessing how digital therapeutics may influence real-world system performance, including resource use, service capacity, and access to care.

**Figure 1.**
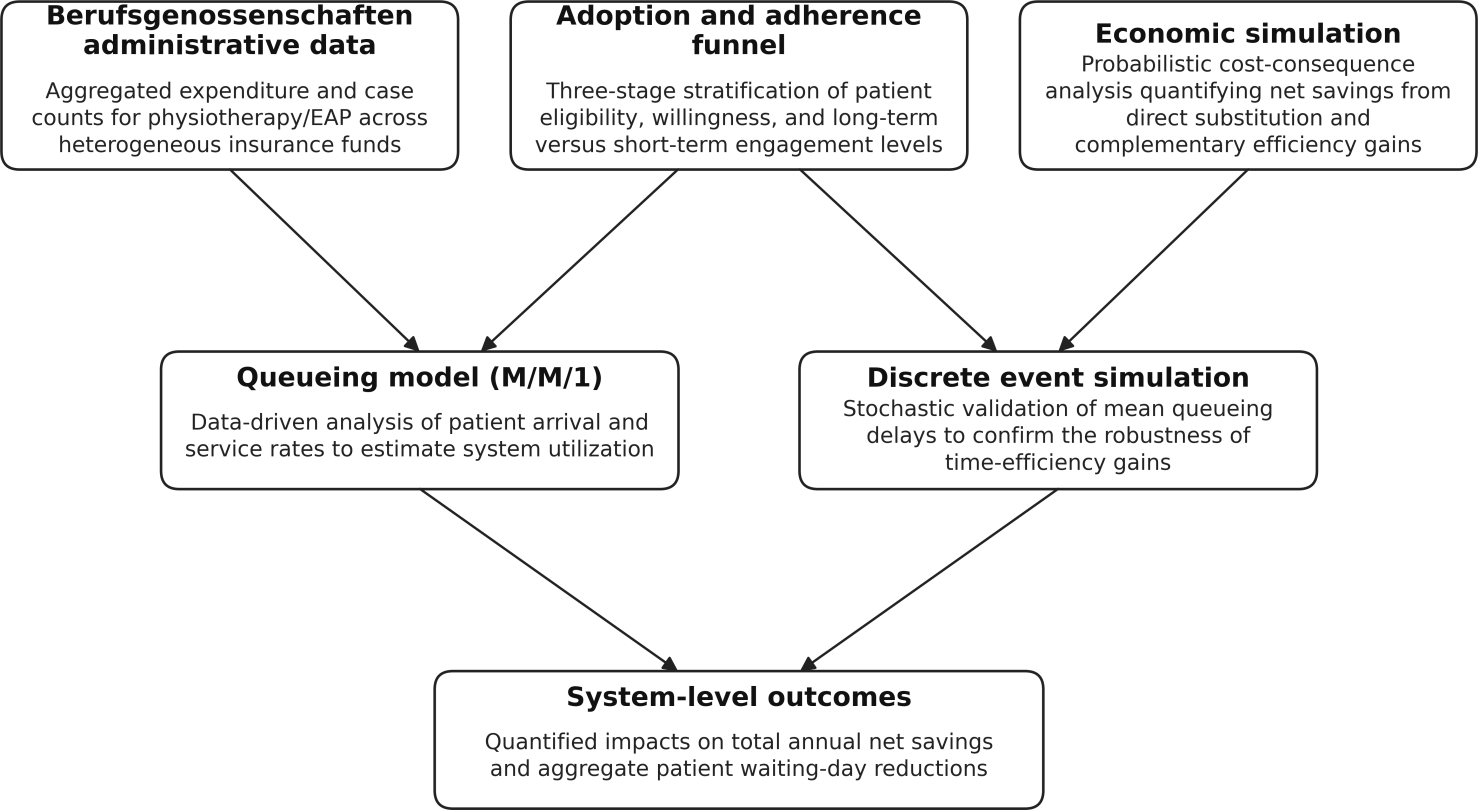
Conceptual framework of the multilevel simulation model evaluating digital health integration into occupational rehabilitation. Administrative data from German statutory accident insurance funds (Berufsgenossenschaften) provide aggregated information on physiotherapy and extended outpatient physiotherapy utilization and expenditures. These data inform an adoption and adherence funnel representing real-world eligibility, uptake, and engagement with digital health applications. Economic and operational effects are estimated using probabilistic simulation modeling substitution and efficiency mechanisms. Changes in therapeutic demand are propagated into a queuing model (M/M/1) to estimate system utilization and waiting-time dynamics, with results validated through discrete event simulation. The integrated framework generates system-level outcomes, including net costs, waiting times, and access-to-care effects. EAP: extended outpatient physiotherapy.

The framework integrates three interconnected components: (1) administrative expenditure data from German statutory accident insurance funds (Berufsgenossenschaften), (2) an adoption and adherence model reflecting real-world patient engagement patterns, and (3) system dynamics modeling using probabilistic simulation and queuing-based capacity analysis. A discrete event simulation (DES) was implemented to validate time-efficiency estimates under stochastic conditions. An overview of the conceptual structure and analytical workflow is shown in Figure 1.

### Data Source and Preparation

We analyzed aggregated, nonpublic administrative summaries from a purposive sample of 5 German statutory accident insurance funds (Berufsgenossenschaften) for fiscal years 2023 to 2024, covering 25.9 million insured individuals. The sample spans heterogeneous risk profiles—for example, Berufsgenossenschaften Bau (construction; high-to-severe trauma exposure) and BGW (health and welfare; higher burden of chronic musculoskeletal conditions)—to improve external validity across occupational settings. For each Berufsgenossenschaften, we obtained annual expenditure and case counts for physiotherapy and EAP, as well as the number of insured employees.

Reporting categories were harmonized to common physiotherapy and EAP definitions using fund fee schedules. Quality checks covered nonnegativity and plausible ranges, reconciliation of physiotherapy and EAP subtotals with Berufsgenossenschaften aggregates, and cross-year consistency. Monetary values are nominal euros (no price adjustment). Missing fields were not imputed; the affected strata were excluded from rate calculations but retained in totals when available. Derived indicators included spend per case, cases per 1000 insured, year-over-year growth, and the arrival-rate proxy used in queuing analysis. The dataset contained no individual-level or identifiable information.

### Economic Modeling Framework

We developed a cost-consequence model from the perspective of the statutory accident insurance funds (reference year: 2024). Net annual savings from DiGA integration into physiotherapy and EAP are the sum of two mutually exclusive mechanisms: (1) direct substitution of conventional therapy with a DiGA and (2) complementary use of a DiGA as an add-on that increases efficiency in nonsubstituted cases. Physiotherapy and EAP are modeled separately and then aggregated.

Model 1 (Direct substitution): A proportion R_Sub_ of conventional cases is fully replaced by a DiGA; savings per therapy type = avoided conventional cost − DiGA cost. Model 2 (Complementary efficiency): Among the remaining (1 − R_Sub_) cases, a share R_Comp_ receives a DiGA add-on, yielding an efficiency gain E_Gain_ (fractional reduction in standard care cost). Net savings per case equal (E_Gain_ × standard care cost) − DiGA cost. By construction, the 2 mechanisms apply to disjoint case partitions, precluding double counting. Adoption and engagement follow the adherence model described below.

### Probabilistic Sensitivity Analysis

To propagate parameter uncertainty, we ran a Monte Carlo probabilistic sensitivity analysis (PSA) with 10,000 iterations. In each iteration, parameters were sampled from their prespecified distributions (Table 1), the model was evaluated for physiotherapy and EAP, and the outputs were aggregated across Berufsgenossenschaften. We report the mean, median, and 95% uncertainty range (2.5th-97.5th percentiles), as well as the probability of any net savings. Heterogeneity was assessed by repeating the PSA per Berufsgenossenschaften to obtain Berufsgenossenschaften-specific means and uncertainty ranges.

**Table 1.** Model parameters and evidence-based probability distributions for the probabilistic sensitivity analysis.

Parameter	Symbol	Description	Distribution	Min, Mode, Max	Source
DiGA^a^ cost	C_DiGA_	Cost for one DiGA prescription per case	Triangular	€250^b^, €450, €600	Goeldner and Gehder [5]
Physiotherapy substitution rate	R_Sub_, physiotherapy	% of physiotherapy cases fully replaced by DiGA	Triangular	5%, 10%, 25%	Informed by digital physiotherapy cost literature [9] and conservative assumptions
EAP^c^ substitution rate	R_Sub_, EAP	% of EAP cases fully replaced by DiGA	Triangular	1%, 3%, 8%	Conservative assumption
Efficiency gain	E_Gain_	Cost reduction per case with DiGA add-on	Triangular	10%, 20%, 30%	Shambushankar et al [11]
Physiotherapy complementary use rate	R_Comp_, physiotherapy	% of remaining physiotherapy cases using complementary DiGA	Triangular	20%, 35%, 50%	Informed by survey-based evidence on digital health adoption [13] and conservative assumptions
EAP complementary use rate	R_Comp_, EAP	% of remaining EAP cases using complementary DiGA	Triangular	15%, 25%, 40%	Conservative assumption

a DiGA: digital health application.

b A currency exchange rate of €1=US $1.18 is applicable.

c EAP: extended outpatient physiotherapy.

### Model Parameters and Distributions

Key parameters follow the substitution and complementary logic and are specified with triangular distributions (minimum, mode, and maximum), informed by recent evidence on the German DiGA market and international digital physiotherapy literature. The exact values and sources are listed in Table 1. All costs are calculated on a per-case basis. Parameter draws are independent unless stated otherwise.

### Patient Adherence Modeling

To enhance the real-world validity of the simulation and reflect that not all patients use DiGA with the same intensity, we integrated an evidence-based patient adherence model (Multimedia Appendix 1). This step is critical, as real-world data from Germany indicate that a significant portion of users discontinue DiGA use after the initial prescription period, and clinical effectiveness is closely linked to the level of patient engagement in a dose-response relationship [14].

The model stratifies the patient population within each simulation run into 2 distinct cohorts based on their adherence level [14]. The first cohort (engaged users) represents patients who exhibit long-term adherence, defined as renewing their DiGA prescription for at least a second cycle. According to real-world claims data from Germany’s largest statutory health insurer, this group comprises approximately 15% based on publicly available German market data [15]. We assume that only patients in this cohort can realize the full potential of the intervention. Accordingly, the literature-based parameters for substitution and efficiency gains were exclusively applied to this group.

The second cohort (short-term users) comprises the remaining 85% of patients who use the DiGA only for the initial prescription period and then discontinue [15]. For this group, we conservatively assumed that their limited engagement is insufficient to produce a measurable clinical or economic effect in terms of substitution or efficiency gains, which were set to zero. However, we modeled a partial benefit equivalent to 3 physiotherapy sessions for short-term users, reflecting front-loaded value delivery within the initial prescription cycle. The full cost of the initial DiGA prescription was applied regardless of adherence status. This adherence-based stratification was applied within each of the 10,000 Monte Carlo iterations.

### Outcome Measures

The primary outcome was total annual net savings (in euros) across the included Berufsgenossenschaften. The secondary outcomes included Berufsgenossenschaften-specific savings and the probability of any savings. Break-even results are reported separately. Analyses were conducted using Python 3.10 (Pandas, NumPy, and Matplotlib). All simulations were executed with fixed random seeds to ensure reproducibility.

### Break-Even Threshold Analysis

With parameters fixed at their mode (base case), we solved two deterministic thresholds: (1) the maximum DiGA price (C_DiGA_) that yields cost neutrality and (2) the minimum engaged adherence rate required for cost neutrality at the base-case DiGA price. These thresholds define operational and commercial boundary conditions for viability in this setting.

### Queuing Model and DES

We quantified the effect of DiGA integration on access and time efficiency by modeling the EAP pathway as an M/M/1 queue (Poisson arrivals, exponential service times, and single server representing total therapeutic capacity). The model is parameterized by the mean arrival rate (*λ*) and service rate (*μ*). *λ* was derived from administrative counts as annual EAP cases divided by 250 business days; μ was obtained by calibration using an empirical waiting-time sample (N=31 patients) from one author’s rehabilitation center. In this framework, the probability of any wait equals system utilization (*ρ*=*λ*/*μ*). We defined a clinically relevant delay as greater than 3 days and used the observed share exceeding this threshold (96.8%) as a data-driven estimator of baseline *ρ*. With *λ* and *ρ* known, *μ* followed algebraically. DiGA scenarios were implemented as proportional reductions in *λ* according to demand changes predicted by the adherence funnel.

The primary end point was the mean waiting time in queue per patient (Wq), which we extrapolated to the annual EAP population to obtain total patient waiting days saved as a system-level measure of improved accessibility. It is important to note that the calibration sample (N=31) is small and originates from a single center. While this provides a data-driven indication of high system use, the exact magnitude of waiting-time reductions may vary across regions and insurance funds. This limitation is inherent to the first iteration of the model and will be addressed in future multicenter extensions.

To validate the analytic M/M/1 results, we implemented DES with Poisson arrivals, exponentially distributed service times, and first-come first-served discipline. Run length and warm-up were chosen to control Monte Carlo error and remove transients: at least 20,000 post–warm-up patients per replication with a 2000-patient warm-up, repeated over 200 independent replications. Building on this calibrated baseline, we compared the status quo model against several DiGA integration scenarios derived directly from the therapeutic demand reduction predicted by the multistage patient selection and adherence funnel: a realistic case (base-case funnel parameters) and conservative and ambitious cases represented 50% and 150% of this effect, respectively.

### Ethical Considerations

This study analyzed aggregated administrative data without individual-level or identifiable information. According to applicable regulations, formal ethical approval and informed consent were not required. All analyses complied with institutional data protection policies and the Declaration of Helsinki.

## Results

### Descriptive Statistics and Cost Dynamics

The analysis used 2024 baseline data from 5 German statutory accident insurance funds—Berufsgenossenschaften Bau (Construction), BGHM (Wood and Metal), BGHW (Trade and Logistics), VBG (Administration), and BGW (Health and Welfare)—covering 25.9 million insured persons. In 2024, the combined expenditures for physiotherapy and EAP across these Berufsgenossenschaften totaled €404.0 million (nominal). Year-over-year, costs rose by 10.1%, while patient volume increased by 1.2%, indicating that spending growth outpaced volume by approximately 9 percentage points. This widening gap signaled mounting financial pressure in rehabilitation pathways and motivated the subsequent simulation of DiGA integration.

### Probabilistic Sensitivity Analysis

**Figure 2.**
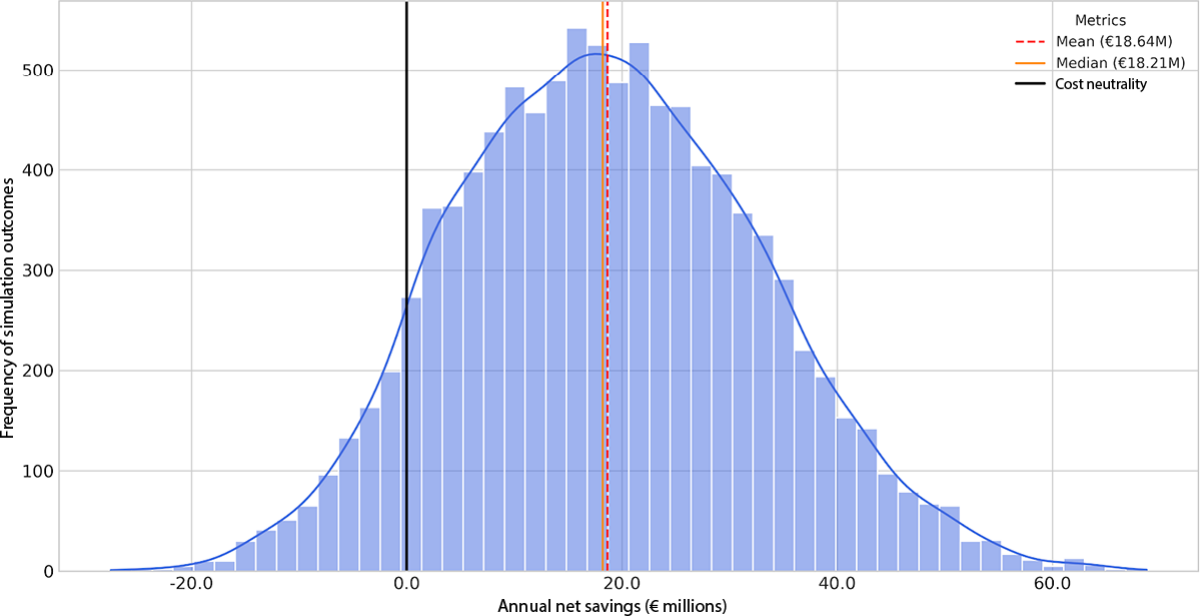
Probabilistic sensitivity analysis of the net financial impact of digital health application (DiGA) integration. The histogram displays the frequency distribution of potential annual net savings resulting from 10,000 Monte Carlo simulation iterations. The model incorporates evidence-based probability distributions for all key parameters, including DiGA costs, substitution rates, efficiency gains, and patient adherence. Vertical lines indicate the mean annual net savings (€18.4 million), the median (€17.9 million), and the cost-neutrality threshold (€0). The significant shift of the distribution's probability mass into the positive range demonstrates the high likelihood (90.7%) of a favorable financial outcome from DiGA integration in the German occupational rehabilitation setting.

The primary simulation, encompassing 10,000 Monte Carlo iterations, quantified the prospective net financial impact of DiGA integration across the German statutory accident insurance landscape. The analysis revealed a mean annual net savings of €18.4 million (median: €17.9 million). The model predicts a 90.7% probability that DiGA adoption will be cost-saving, with a 95% uncertainty range for the annual financial impact spanning from a net cost of €8.0 million to a net saving of €47.7 million. Figure 2 displays the full probability distribution of the 10,000 simulated outcomes, illustrating a significant rightward shift of the probability mass relative to the cost-neutrality threshold.

### Heterogeneity of Financial Impact Across Insurance Funds

**Figure 3.**
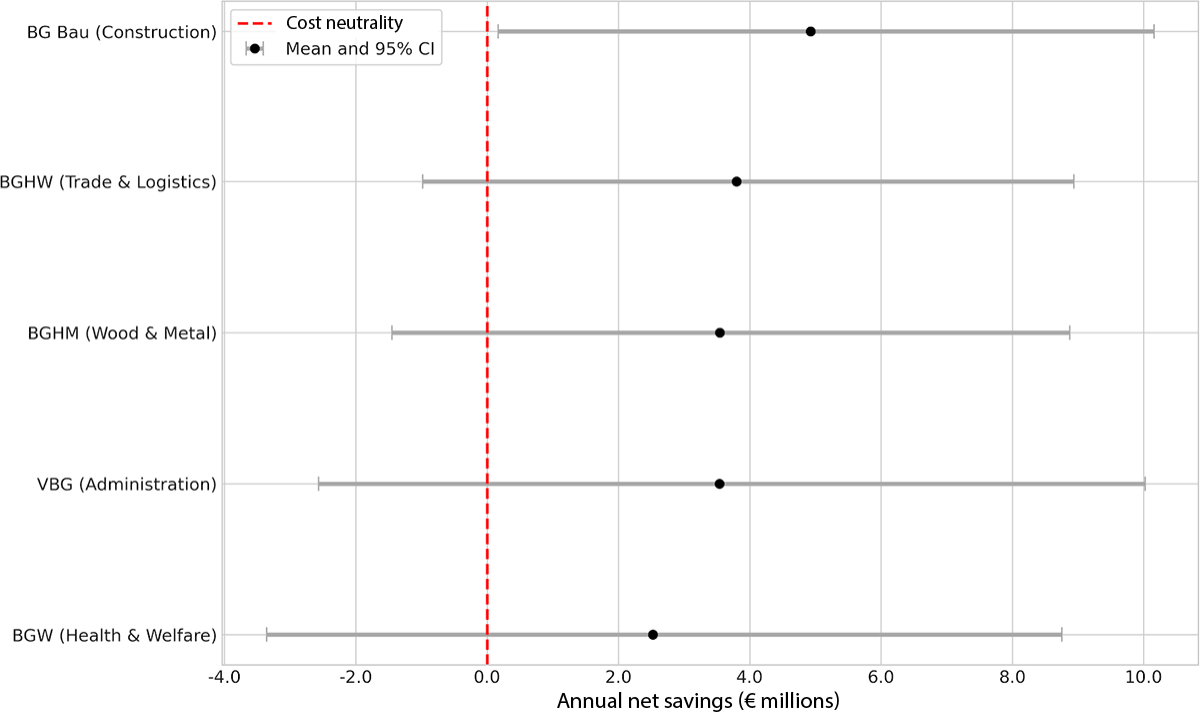
Heterogeneity of financial impact across Berufsgenossenschaften. The forest plot displays the mean annual net savings (points) and the corresponding 95% uncertainty range (horizontal lines) for each of the 5 analyzed German statutory accident insurance funds (Berufsgenossenschaften). Results are derived from a stratified probabilistic sensitivity analysis with 10,000 Monte Carlo iterations per Berufsgenossenschaften. The vertical dashed line at €0 represents the cost-neutrality threshold. The plot illustrates the differential impact of DiGA integration, with the highest point estimate for savings found in the Berufsgenossenschaften serving the construction sector (Berufsgenossenschaften Bau).

To investigate whether the potential for cost savings varied across different patient populations, the PSA was stratified by each individual Berufsgenossenschaften. This analysis revealed substantial heterogeneity in the estimated financial impact (Figure 3). While all analyzed Berufsgenossenschaften demonstrated a positive mean net saving, the magnitude and certainty of savings differed. Berufsgenossenschaften Bau, which covers the construction sector with a high incidence of severe traumatic injuries, showed the highest mean annual net savings at €4.9 million (95% CI €0.2-€10.3 million). Conversely, Berufsgenossenschaften covering administrative sectors (VBG) and health and welfare services (BGW), which often manage more chronic conditions, showed a smaller, though still positive, mean financial benefit. Notably, the 95% uncertainty ranges for these latter Berufsgenossenschaften overlap with the cost-neutrality threshold, indicating a higher degree of uncertainty regarding the financial outcome in these specific patient populations.

This heterogeneity likely reflects differences in baseline cost structures, injury severity profiles, and the relative share of acute versus chronic rehabilitation pathways across sectors. Construction sector rehabilitation typically involves high-cost posttraumatic pathways where substitution effects generate larger absolute savings, whereas administrative and health care sectors may have lower per-case costs and different rehabilitation patterns. These findings suggest that the economic return on DiGA integration is not uniform but rather dependent on the sectoral composition of the insured population and the cost intensity of prevailing rehabilitation pathways.

### Patient Selection and Adherence Funnel

To generate the most realistic forecast of the net financial impact, we applied a 3-stage patient selection and adherence funnel to the simulation (Multimedia Appendix 1). This final model assumes that only 70% of potential candidates are eligible for and accept a DiGA prescription. This cohort is then stratified into long-term engaged users (15%) and short-term users (85%), with the latter achieving a partial benefit equivalent to 3 physiotherapy sessions.

**Figure 4.**
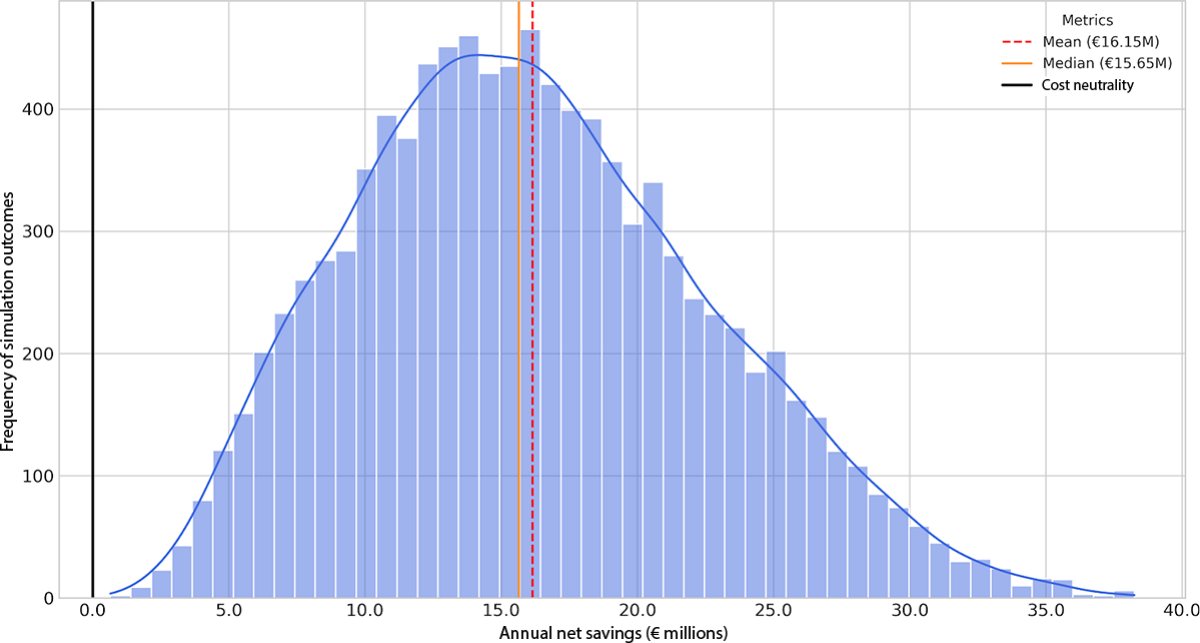
Net financial impact of digital health application integration under a multistage patient selection and adherence funnel model. The histogram displays the frequency distribution of net savings from 10,000 Monte Carlo iterations. The model incorporates a 70% patient eligibility and acceptance filter, followed by a stratification into long-term (15%) and short-term (85%) users, with the latter achieving a partial benefit. The entire probability mass is located in the positive range, indicating a 100% probability of a cost-saving outcome, with a mean net saving of €16.2 million.

This refined analysis yields a robustly positive financial case for DiGA integration. The simulation forecasts a mean annual net saving of €16.2 million (median: €15.7 million). Critically, the entire 95% uncertainty range (€5.0-€29.8 million) lies in positive territory, resulting in a 100% probability of achieving a net positive financial outcome (Figure 4). This demonstrates that even after applying conservative filters for patient eligibility and accounting for suboptimal real-world adherence patterns, the integration of DiGA remains financially viable.

Notably, the structural dependency on partial benefits from short-term users represents a key finding of the adherence analysis. The break-even threshold analysis (see *Break-Even Threshold Analysis* section) confirms that even under maximally optimistic assumptions regarding long-term adherence, the model cannot achieve cost neutrality without capturing value from the initial prescription cycle of short-term users. This underscores that economic sustainability in digital therapeutics is not solely a function of durable adherence but critically depends on early-cycle benefit realization—a finding consistent with evidence on high attrition and the importance of early engagement in digital health interventions [16].

### Break-Even Threshold Analysis

**Figure 5.**
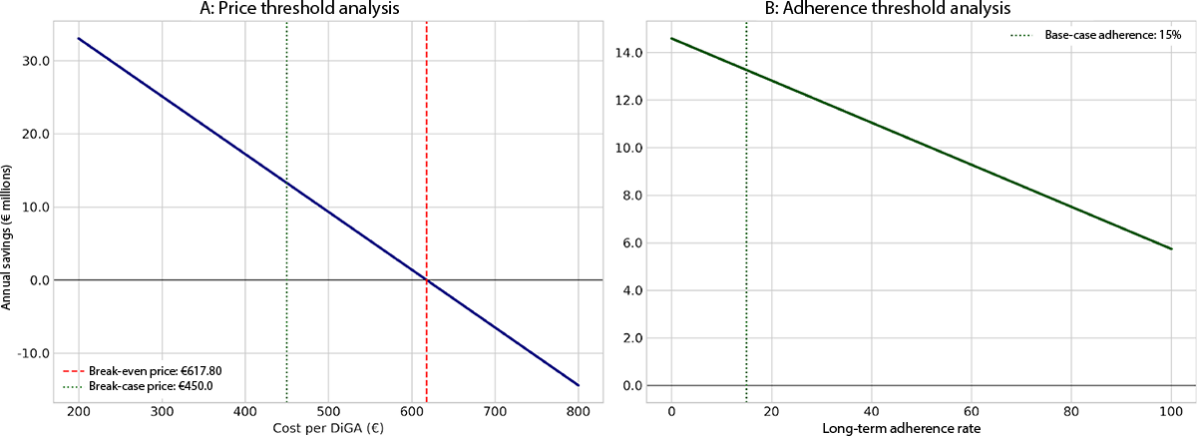
Deterministic break-even analysis for DiGA price and patient adherence. The figure displays 2 one-way sensitivity analyses based on the final funnel model with all other parameters held at their base-case values. (A) The effect of varying the cost per DiGA on annual net savings. The model breaks even at a maximum price of €617.80. (B) The effect of varying the long-term adherence rate on annual net savings. The model fails to break even within the plausible 0% to 100% adherence range, indicating that the partial benefit from short-term users is essential for the overall implementation feasibility. DiGA: digital health application.

To determine the commercial and operational thresholds for implementation feasibility, a deterministic break-even analysis was conducted using the base-case parameters of the final funnel model (Figure 5). The analysis of the price threshold (Figure 5A) shows that DiGA integration remains cost-saving for an average DiGA price of up to a maximum of €617.80. This indicates that even at a price point nearly 40% higher than the base-case assumption of €450, the intervention does not result in a net loss, demonstrating substantial economic robustness. The model further showed that the long-term adherence rate alone cannot drive cost neutrality (Figure 5B): the calculated break-even adherence rate of 164.8% is a mathematical impossibility, confirming that the partial benefit from the 85% of short-term users is structurally indispensable for overall implementation feasibility.

### System-Level Impact on Care Accessibility

**Figure 6.**
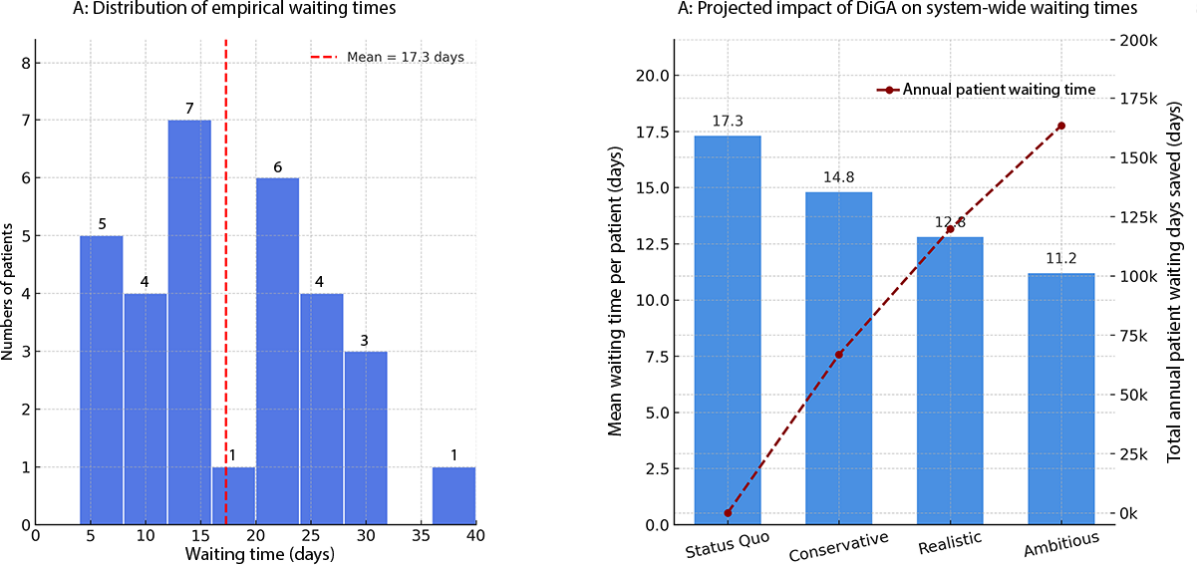
Impact of digital health application (DiGA) integration on extended outpatient physiotherapy (EAP) waiting times, based on a data-driven queuing model. The figure displays the results of the system-level time-efficiency analysis. (A) The frequency distribution of empirical waiting times from a sample of patients (N=31). The proportion of patients waiting more than 3 days was used to calibrate the queuing model to a baseline system utilization of 96.8%, which accurately reproduces the observed mean waiting time of 17.3 days. (B) A dual-axis chart showing the projected impact of different DiGA-induced demand reduction scenarios. The bars (left axis) represent the mean waiting time per patient, which is projected to decrease from 17.3 days to 12.8 days in the realistic scenario. The dashed line (right axis) shows the total annual patient waiting days saved for the entire EAP patient population (26,705), culminating in approximately 120,000 saved days in the realistic scenario. DiGA: digital health application.

To quantify the impact of DiGA integration on time efficiencies, we modeled the EAP care pathway as a queuing system. The model was calibrated directly using empirical patient waiting-time data (N=31 patients), as shown in Figure 6A. We defined system use (ρ) as the proportion of patients waiting more than a clinically reasonable period of 3 days for therapy initiation. This data-driven approach revealed a mean system use of 96.8% based on the cohort, which is consistent with operation near capacity in the sampled setting and is therefore highly sensitive to changes in patient demand.

The simulation of DiGA integration under this calibrated model forecasts a substantial reduction in systemic waiting times (Figure 6B). The status quo scenario reproduces the empirically observed mean waiting time of 17.3 days per patient. In a realistic scenario, which assumes a modest reduction in therapeutic demand based on the final funnel model, the mean waiting time per patient decreases by 26% to 12.8 days. When extrapolated to the entire EAP patient population (26,705 patients), this reduction corresponds to a system-wide saving of approximately 120,000 cumulative patient waiting days annually. This finding indicates that in a capacity-constrained environment, DiGA can act as a critical release valve, mitigating systemic bottlenecks and increasing patient access to care.

For validation, results were corroborated in a DES parameterized to the same arrival and service rates. Under the calibrated baseline, the DES yielded a mean waiting time of approximately 17.3 days (95% uncertainty range: 15.5‐19.6 d). A DiGA scenario with a 15% demand reduction produced a mean waiting time of approximately 10.58 days (95% range: 9.7‐11.7 d), confirming the magnitude and direction of the analytic M/M/1 estimates (Multimedia Appendix 2).

### Estimated Productivity Implications

To provide a preliminary estimate of indirect cost implications, we translated the projected waiting-time reductions into productivity-related outcomes. If the 120,000 cumulative patient waiting days saved annually correspond even partially to accelerated therapy initiation, the downstream implications for return-to-work timelines are substantial. Using publicly available data on average gross daily wages in the German construction sector (approximately €180 per business day) and accounting for Verletztengeld (injury-related wage replacement benefits, typically 68%‐80% of net wages), a conservative extrapolation suggests that the productivity-related value of reduced waiting could reach several million euros annually across the insured population, though precise quantification requires patient-level linkage data not available in this study. This estimate is intended as an order-of-magnitude indication rather than a definitive calculation and underscores the need for prospective studies that capture indirect costs alongside direct rehabilitation expenditures.

## Discussion

### Principal Findings

This study presents a multilevel simulation demonstrating that the integration of digital therapeutics into Germany’s occupational rehabilitation system is likely to generate substantial economic benefits and system-level capacity gains under conservative, real-world assumptions. The primary simulation indicated mean annual net savings of €18.4 million (90.7% probability of cost savings), which increased to €16.2 million with a 100% probability of positive financial impact after incorporating adherence dynamics. Cost neutrality was maintained for DiGA prices up to €617.80 per prescription—nearly 40% above the base-case assumption—indicating substantial economic robustness. A calibrated queuing model projected a 26% reduction in mean EAP waiting times, equivalent to approximately 120,000 patient waiting days saved annually, demonstrating DiGA’s potential as a strategic capacity tool in addition to its cost-saving properties.

### Germany as a Real-World Laboratory for Digital Therapeutics

As the first country to establish a formal reimbursement pathway for digital therapeutics, Germany offers a unique real-world laboratory for evaluating digital medicine at scale [17]. By anchoring our model in actual cost data from Germany’s statutory accident insurance funds and leveraging claims-based adherence rates, this work moves beyond theoretical potential to a more realistic forecast. The finding that the probability of a positive financial return approached 100% after adherence adjustment suggests that the economic case for DiGA integration is not merely marginal but substantial.

### The Decisive Role of a Structured Implementation Pathway

Perhaps the most significant methodological contribution of this work is the shift from a simplistic universal-provision model to an evidence-based, multistage patient selection and adherence funnel. This approach underscores that the implementation feasibility of DiGA is not an inherent feature of the technology alone, but rather a function of the care pathway in which it is embedded. The model demonstrates that digital therapeutics cannot be distributed indiscriminately with an expectation of positive returns. Instead, it highlights the necessity of a structured implementation pathway that accounts for patient eligibility, clinical triage, and real-world engagement patterns, highlighting the importance of patient selection and sustained engagement in digital health interventions [18,19].

### Heterogeneity of Impact and Precision Reimbursement

The stratified analysis reveals that the financial benefit of DiGA is not uniformly distributed across patient populations. The observation that savings potential is highest in Berufsgenossenschaften Bau (construction sector) implies that DiGA may deliver the greatest value when applied to acute, high-cost, posttraumatic rehabilitation pathways. This heterogeneity challenges current generic approaches to prescribing and reimbursement and suggests that future policy iterations should consider precision reimbursement models with value-based pricing structures that incentivize DiGA application in the highest-need, highest-cost cohorts [20-22]. The differential impact across sectors further highlights that the economic case for DiGA should be evaluated at the level of specific rehabilitation pathways rather than as a blanket system-wide metric.

### Economic Boundaries as a Policy Signal

The break-even analysis delineates the boundary conditions for DiGA integration. The model demonstrates substantial robustness to price variation: cost neutrality persists until an average prescription price of €617.80, nearly 40% above the reference case (€450), aligning with recent evaluations of the German DiGA reimbursement framework [5]. In sharp contrast, the analysis shows that partial benefits from short-term users are indispensable for cost-effectiveness. Even under maximally optimistic assumptions, higher long-term adherence alone cannot achieve break-even within our model; without capturing value from short-term engagement, the model remains structurally loss-making. This counterintuitive finding underscores that economic sustainability is not solely a function of durable adherence but critically depends on early-cycle benefit realization, consistent with evidence on digital health attrition and front-loaded value delivery [16]. For policymakers, this implies that price negotiation and reimbursement design may be a more effective lever than adherence-focused interventions alone. For developers, it highlights the strategic imperative of creating interventions that deliver measurable benefits within the first prescription cycle and remain effective despite predictable attrition in digital health interventions [19].

### System-Level Capacity Effects

The queuing analysis reveals a potentially profound impact, demonstrating DiGA’s capacity to resolve systemic bottlenecks in care delivery. Calibrating the queuing model against empirical data showed that 96.8% of sampled patients experienced waits exceeding 3 days, consistent with near-capacity operation. Under queuing theory, such conditions predict highly nonlinear effects: the projected 26% reduction in mean waiting time in the realistic scenario—from 17.3 to 12.8 days—and the system-wide saving of approximately 120,000 cumulative patient-days annually underscore this disproportionate improvement resulting from a modest reduction in demand. Consistency with the DES further supports these findings: the DES reproduced the calibrated baseline delay and projected a comparable reduction, demonstrating robustness to stochastic variation. Taken together, analytic and simulated results indicate that modest, targeted demand reductions can produce outsized accessibility gains once the system is nudged below its critical utilization threshold. This suggests that DiGA should be viewed not merely as a cost-saving substitute but as a strategic instrument to generate capacity and enhance patient access.

### Implications for Global Digital Health Policy

The findings offer actionable insights beyond Germany. For policymakers in other countries, the model provides a clear economic rationale for investing in digital therapeutic frameworks [23,24], with the critical caveat that these investments must be coupled with strategies to manage patient selection and support engagement. For the global digital health industry, the results highlight that the central challenge is not only demonstrating clinical efficacy but also solving the real-world problem of patient adherence [16,18,19]. Ultimately, the most significant system-level impact of effective digital rehabilitation may extend beyond direct savings. The estimated reduction of approximately 120,000 waiting days annually could translate into substantially earlier return-to-work timelines, mitigating the indirect costs of lost productivity that constitute the largest component of the total economic burden of occupational injuries. The ultimate value of DiGA should therefore be contextualized not only by direct savings for insurers but also by their potential contribution to reducing the macroeconomic burden of workplace accidents [23,24].

### Limitations

As a simulation-based implementation study, several limitations should be considered. First, the predictive accuracy of the simulation is contingent upon the validity of its input parameters. While grounded in the best available evidence, key assumptions—particularly the DiGA substitution rate (R_Sub) and the efficiency gain (E_Gain)—are derived from international studies and clinical equivalence trials rather than direct real-world data from the German occupational insurance system. The assumed substitution of complex, multimodal EAP cases, though modeled conservatively, remains especially theoretical. Consequently, the calculated savings should be interpreted as potential estimates under specific evidence-based assumptions, not as a definitive forecast. This reliance on literature-derived parameters is, however, standard practice in simulation modeling for health technologies at early adoption stages, where direct real-world data are not yet available, and the robustness of findings is ensured through a 10,000 iteration PSA.

The adherence model, while innovative in its use of real-world German claims data, simplifies a complex behavioral spectrum into a binary engaged versus short-term cohort. The approximately 15% long-term engagement rate, though derived from a large insurer, may not be perfectly generalizable to the Berufsgenossenschaften population.

The queuing model, while providing strong evidence of nonlinear capacity effects, relies on calibration from a small empirical waiting-time sample (N=31 patients) obtained from a single rehabilitation center. Although this sample provides a data-driven indication of high system use, it limits the precision of absolute waiting-time estimates and may not fully represent regional or fund-specific variation. Future studies should aim to incorporate larger, multiregional datasets and multiserver capacity dynamics. Additionally, while the DES validates the analytic model, this first iteration shares the single-server and exponential-service assumptions; extensions to multiserver capacity, empirical service-time distributions, day-specific capacity calendars, and no-show dynamics are planned to stress-test generalizability.

Finally, the analysis is based on aggregated data, precluding stratification by patient-level factors (diagnoses, comorbidities, age) known to influence outcomes and technology uptake.

### Future Directions

While this study models the financial dimension of DiGA integration, a holistic valuation requires further analysis of its impact on care capacity and indirect costs. The next step is a richer DES incorporating multiserver capacity, weekday calendars, and empirical duration and no-show distributions calibrated to larger real-world datasets. To progress from forecasting toward causal inference, findings should be validated with prospective, patient-level data. A randomized controlled trial or rigorously adjusted observational study within the German Berufsgenossenschaften system would be the gold standard. Finally, implementation science studies should focus on clinical decision support for eligibility and triage and on evidence-based engagement strategies that sustain long-term use.

## Supplementary material

10.2196/93793 Multimedia Appendix 1 Conceptual flowchart of the multistage patient selection and adherence funnel. This diagram visually outlines the 3-stage model used to simulate the patient journey and its economic consequences under DiGA integration. The process begins with stage 1: patient eligibility and willingness, where a “pool of potential patients” undergoes a clinical and preference-based triage. Only those deemed “eligible & willing” (70%) proceed to DiGA prescription; others remain in the “standard care pathway.” In stage 2: patient adherence profile, the prescribed cohort is stratified based on real-world adherence patterns into “short-term user cohort” (85%) and “engaged user cohort” (15%). Finally, stage 3: outcome assigns differential benefits: “short-term users” achieve a partial economic benefit, while “engaged users” realize the full potential benefit, reflecting a dose-response relationship between engagement and therapeutic outcome.

10.2196/93793 Multimedia Appendix 2 EAP waiting time (DES): baseline vs DiGA scenario. Bars display the DES estimate of the mean queuing delay per patient E[Wq] in days for the calibrated status quo system and for a DiGA integration scenario that reduces demand by 15% (λ′=0.85 λ). Using 2025 EAP starts (27 cases; 250 business days), we set λ=0.108 day−1; the service rate was calibrated to observed waiting times, yielding μ≈0.150 day−1. The DES reproduces the empirical mean delay at baseline (mean 17.28 days; 95% replication range 15.69-19.42) and projects a shorter delay with DiGA (mean 10.51 days; 95% 9.79-11.67). The implied system-level effect corresponds to a substantial reduction in accumulated waiting time across the annual EAP cohort.

## Abbreviations

DES: discrete event simulation
DiGA: digital health application
EAP: extended outpatient physiotherapy
PSA: probabilistic sensitivity analysis
