# Supplementary figures and images for: Integration of Digital Therapeutics Into Occupational Rehabilitation in Germany: Multilevel Simulation Study
Source: J Med Internet Res. 2026 May 26;28:e93793. doi: 10.2196/93793 (PMC13211607; doi:10.2196/93793)

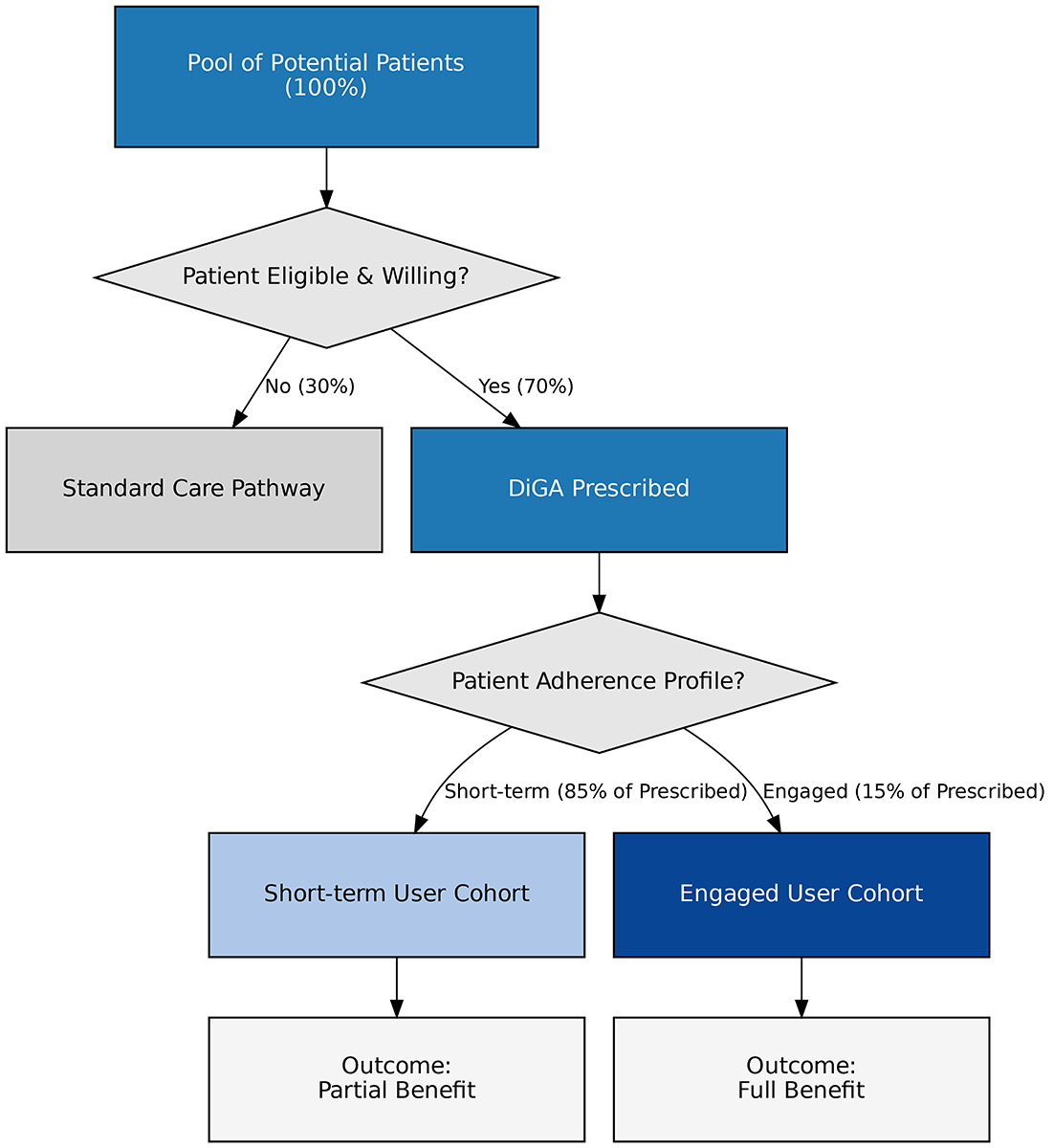

Supplement: Multimedia Appendix 1 [file jmir-v28-e93793-s001.png]

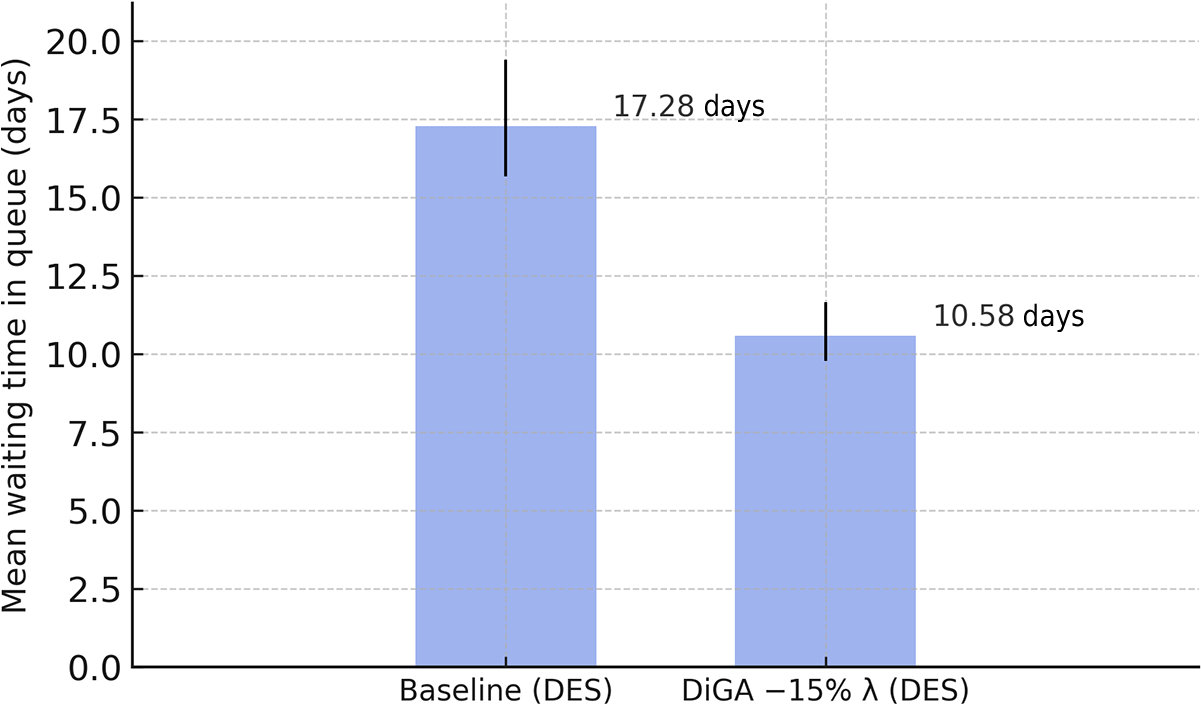

Supplement: Multimedia Appendix 2 [file jmir-v28-e93793-s002.png]
